# Stroke awareness and knowledge in Sudan: a cross-sectional analysis of public perceptions and understanding

**DOI:** 10.3389/fpubh.2024.1362979

**Published:** 2024-05-01

**Authors:** Eyman M. Eltayib, Feras Jirjees, Duaa Suliman, Hala AlObaidi, Munazza Ahmed, Zelal J. Kharaba, Yassen Alfoteih, Muna Barakat, Zainab Khidhair, Husam ALSalamat, Nazik Mustafa, Sarah Cherri, Sami El Khatib, Souheil Hallit, Diana Malaeb, Hassan Hosseini

**Affiliations:** ^1^College of Pharmacy, Jouf University, Sakaka, Saudi Arabia; ^2^College of Pharmacy, University of Sharjah, Sharjah, United Arab Emirates; ^3^Health Policy, Mohammed Bin Rashid School of Government, Dubai, United Arab Emirates; ^4^School of Pharmacy, Queen's University Belfast, Belfast, United Kingdom; ^5^College of Pharmacy, Al Ain University, Abu Dhabi, United Arab Emirates; ^6^College of Dentistry and College of Humanities, City University College of Ajman, Ajman, United Arab Emirates; ^7^Faculty of Pharmacy, Applied Science Private University, Amman, Jordan; ^8^College of Science, University of Baghdad, Baghdad, Iraq; ^9^Faculty of Medicine, Al-Balqa Applied University, Al-Salt, Jordan; ^10^Department of Pharmacology, Faculty of Pharmacy, Al Neelain University, Khartoum, Sudan; ^11^Lebanese International University, School of Pharmacy, Beirut, Lebanon; ^12^Department of Biomedical Sciences, Lebanese International University, Bekaa, Lebanon; ^13^Center for Applied Mathematics and Bioinformatics (CAMB), Gulf University for Science and Technology, West Mishref, Kuwait; ^14^School of Medicine and Medical Sciences, Holy Spirit University of Kaslik, Jounieh, Lebanon; ^15^Applied Science Research Center, Applied Science Private University, Amman, Jordan; ^16^College of Pharmacy, Gulf Medical University, Ajman, United Arab Emirates; ^17^UPEC-University Paris-Est, Creteil, France; ^18^RAMSAY SANTÉ, HPPE, Champigny-sur-Marne, France

**Keywords:** stroke, Sudan, knowledge, risk factor, awareness, source of information

## Abstract

**Introduction:**

Stroke, a leading cause of morbidity and mortality globally, demands heightened awareness and knowledge for effective preventive strategies and tailored response. Sudan is classified as a low income country with a low rate of literacy, lack of knowledge, and awareness about diseases. Thus, this study aimed to assess stroke awareness and knowledge among Sudanese population, and identify the associated factors influencing awareness.

**Methods:**

A cross-sectional study conducted between October and November 2022 through a self-administered online survey distributed via various social media platforms. The study involved adults aged 18 years and above through snow-ball sampling technique. The survey covered general awareness and knowledge concerning stroke risk factors, consequences, and the appropriate responses taken during acute stroke attacks.

**Results:**

A total of 410 participants were enrolled in the study, majority (93.4%) were from urban area and had university degree (92.4%). Furthermore, 92.2% were aware about stroke and 74.9% were able to recognize the symptoms of stroke. Only 40.2% identified all correct answers, 96.3, 92.3, and 95.1% recognized at least one risk factor, early symptom, and consequences, respectively. Females were significantly more than males able to identify at least one risk factor. Almost all participants (99.5%) perceived stroke as a serious disease (99.5%). Notably, 86.3% would promptly transport a suspected stroke patient to the hospital. The multivariable analysis showed that females versus males and patients with depression versus without depression had significantly higher odds to identify at least one risk factor (OR of 14.716 [95% CI 1.901; 113.908] and 0.241 [95% CI 0.059; 0.984], respectively).

**Conclusion:**

The study concluded that stroke knowledge and awareness among Sudanese population is suboptimal. Furthermore, early stroke recognition and intake of the appropriate management strategies are lacking which highlights the need for targeted education and awareness campaigns.

## Introduction

Stroke is a major cause of disability and one of the leading causes of death worldwide (1). It is typically defined as a neurological deficit pertaining to an acute focal injury of the central nervous system (CNS) via vascular causes, which include cerebral infarction, intracerebral hemorrhage, and subarachnoid hemorrhage (2). The most common risk factors for stroke include hypertension, impaired cardiac function, smoking, poor lifestyle factors, dyslipidemia, obesity, diabetes, and having a family history of stroke (3). The majority of ischemic stroke occurs in low- and middle-income countries, where incidents of fatal hemorrhage and ischemic strokes develop more often at younger ages. Additionally, compared to developed countries, the prevalence of stroke has recently increased in developing nations (4).

Sudan, a least developed country according to the United Nations, had about 25% of stroke admissions among patients older than 60 years old (5). Furthermore, stroke was reported to be among the top 25 causes of premature death in Sudan between 1990 and 2019 (6). Moreover, statistics from the World Health Organization (WHO) in 2019 show that stroke was the third-top cause of death in both genders, with 48.09 deaths per 100,000 people, in addition to 48,595 new stroke cases recorded in 2019 (7).

Unfortunately, stroke risk factors were found to be higher in poor socioeconomic level societies. Nonetheless, community stroke awareness programs are one of the several components that is considered absolutely necessary for stroke care (8). The outcome of stroke can be greatly improved through early detection, prompt transfer to medical care, and implementation of appropriate medical therapy (9). However, preventive approaches mostly revolve around understanding stroke risk factors, identifying the warning signs, and treating the concomitant conditions. According to the World Bank classification, Sudan is a low-income country (10). In addition, the literacy rates in Sudan is low, particularly among young women (11). With the adult literacy rate of 60.7% (12), identifying the existing gaps in knowledge among Sudanese population regarding stroke can help recognize areas to be targeted to improve overall public health and healthcare practices. Additionally, increased understanding about stroke has been linked with high income status, smoking history, and educational attainment (13). Therefore, it is essential to screen for socioeconomic status, educational achievement, and lifestyle factors.

Comprehensive research focusing on the factors associated with knowledge and awareness of stroke among Sudanese population is extremely essential as it allows the design for targeted interventions and educational campaigns tailored to the unique challenges faced by the population. This research seeks to provide valuable insights into the importance of enhancing stroke knowledge and awareness, aiming to minimize the occurrence of strokes, preventing recurrence, and ensuring early patient recognition. We intend to expand upon existing knowledge by examining a cohort of participants across Sudan. Additionally, the findings from this study could potentially influence the development and implementation of effective interventions, drawing upon reliable population-based data. Thus, the study aimed to evaluate the awareness and knowledge of stroke within the general population in Sudan and identify factors influencing this awareness.

## Method

### Study design and period

A cross-sectional study conducted between October and November 2022 through a self-administered closed-ended online survey designed for the general Sudanese population. The study involved adults aged 18 years and above through snow-ball sampling technique. The survey covered general awareness and knowledge concerning stroke risk factors and consequences. The survey adhered to the general principles of good survey design (14). The survey was developed on Google form and the link was circulated through online platforms like Facebook® and WhatsApp® Participation in the study was voluntary, and eligibility criteria included individuals aged 18 and above, with exclusion of those with a history of stroke. The survey, conducted in the Arabic language, the native language of Sudanese citizens and typically took approximately 10 min to be completed.

### Validation of the survey

The questionnaire was similarly structured to a survey used in a study conducted in Jordan (15). Three academic professionals reviewed the questionnaire. Then the survey underwent a six-person with no medical background pilot test to ensure the clarity of the questions. Subsequently, the questions were modified based on their feedback. The first section of the questionnaire covered the socio-demographic data. The second section assessed the overall knowledge about stroke and evaluated awareness about stroke risk factors, consequences of stroke, and response when facing somebody with a stroke attack. Moreover, it examined knowledge of early warning signs and score of one point was awarded per correct answer to the above statements. The third section identified sources of information related to stroke among the participants.

### Sample size calculation

The target sample size was estimated to be 385 participants using the Raosoft® software sample size calculator (16). This calculation was determined for the minimum sample size required for an unlimited population size, using a 95% confidence interval, a standard deviation of 0.5, and a margin of error of 5%.

### Ethical approval

The study got ethical approval from the research ethics committee of the Faculty of Pharmacy, Al Neelain University, Khartoum, Sudan (NPH1021). All participants agreed to participate in the study by selecting “I agree” on the electronic informed consent form before filling out the questionnaire. All methods were performed in accordance with the relevant guidelines and regulations or declaration of Helsinki.

### Statistical analysis

The data obtained were subjected to analysis using the Statistical Package for the Social Sciences (SPSS) version 27.0. Continuous variables were presented as mean ± standard deviation (SD) with a 95% confidence interval (CI). Categorical and ordinal variables were reported as frequencies and percentages. Logistic regression was performed to identify factors linked to the ability to automatically recognize one or more stroke risk factors, warning signs, consequences, and the inclination to promptly seek emergency room care upon experiencing a stroke. Variables demonstrating a significance level of *p* < 0.2 in the bivariate analysis were incorporated into the regression analysis. Results were presented as odds ratios (OR) with corresponding 95% CI. All statistical tests were two-tailed, and a *p*-value of <0.05 was considered statistically significant.

## Results

### Sample description

From the total of 410 Sudanese participants enrolled in the study; 195 (47.6%) were females, 149 (36.6%) were under 30 years of age, almost half of the participants (51.2%) were married, majority were in urban areas (93.4%), and had university degree (92.4%). More than two thirds of the participants (71.1%) were from the capital city “Khartoum,” in addition, more than quarter (28.3%) were from other regions of Sudan. The most common chronic diseases among the participants were hypertension (26.8%), dyslipidemia (19.8%) and diabetes mellitus (17.3%). The sociodemographic factors displayed in Table 1.

**Table 1:** Participants’ socio-demographic characteristics, past medical history and familiarity with stroke (*n* = 410).

Variables		Frequency (%)
Socio-demographic characteristics		
Gender	Male	215 (52.4%)
	Female	195 (47.6%)
Age groups (years)	Less than 30 years	149 (36.3%)
	Between 30–49 years	199 (48.5%)
	More than 50 years	62 (15.1%)
Residence area	Urban	383 (93.4%)
	Rural	27 (6.6%)
Living place	Khartoum (the capital)	294 (71.7%)
	Central states	44 (10.7%)
	Eastern states	17 (4.1%)
	Northern states	29 (7.1%)
	Western states	26 (6.3%)
Marital status	Single	181 (44.1%)
	Married	210 (51.2%)
	Divorced/Widowed	19 (4.6%)
Education level	School	31 (7.6%)
	University	379 (92.4%)
Employment status	Unemployed	117 (28.5%)
	Employed	293 (71.5%)
Income level*	Low	74 (18.0%)
	Medium	156 (38.1%)
	High	180 (43.9%)
Smoking status	Yes	158 (38.5%)
Past medical history	Hypertension	110 (26.8%)
	Diabetes Mellitus	71 (17.3%)
	Dyslipidemia	81 (19.8%)
	Heart diseases	57 (13.9%)
	Kidney disease	34 (8.3%)
	Gastro problems	61 (14.9%)
	Depression	40 (9.8%)
	Obesity	63 (15.4%)
Familiarity with stroke	Ever heard of stroke	378 (92.2%)
	History of stroke in the family	134 (32.7%)
	Personally know someone with stroke	307 (74.9%)

*Counted based on Sudanese income per month: (low income less than 20,000 Sudanese pounds, Medium income between 20,000 and 50,000 Sudanese pounds, and high income more than 50,000 Sudanese pounds).

### Respondents’ general knowledge about stroke

Table 2 shows analysis of correct responses among the participants related to general knowledge, and identification of risk factors, early symptoms, and consequences of stroke. Most participants had heard of stroke (92.2%), around three quarters (74.9%) knew about stroke if a patient developed the disease. The majority (97.3%) had at least two correct answers regarding general knowledge of stroke. However, 40.2% of the participants could identify all correct answers regarding general knowledge of stroke. In addition, most of the participants were able to correctly identify at least one stroke risk factors, early symptoms, and consequences of stroke with 96.3, 92.3, and 95.1%, respectively. However, 34.4% of the participants identified all the risk factors, 42.7% recognized all the symptoms, and more than half of the participants (53.9%) stated all possible consequences of stroke.

**Table 2:** Number of stroke risk factors, early symptoms, and consequences that were identified by the participants (*n* = 410).

		Frequency (%)	Cumulative, frequency (%)
Number of correct answers regarding general knowledge of stroke	Less than two	11 (2.7)	11 (2.7)
	Two	26 (6.3)	37 (9.0)
	Three	55 (13.4)	92 (22.4)
	Four	153 (37.3)	245 (59.8)
	Five	165 (40.2)	410 (100)
Number of identified risk factors of stroke	Zero	15 (3.7)	15 (3.7)
	One	8 (2.0)	23 (5.6)
	Two	3 (0.7)	26 (6.3)
	Three	10 (2.4)	36 (8.8)
	Four	14 (3.4)	50 (12.2)
	Five	20 (4.9)	70 (17.1)
	Six	32 (7.8)	102 (24.9)
	Seven	41 (10.0)	143 (34.9)
	Eight	56 (13.7)	199 (48.5)
	Nine	70 (17.1)	269 (65.6)
	Ten	141 (34.4)	410 (100)
Number of identified early symptoms of stroke	Zero	31 (7.6)	31 (7.6)
	One	5 (1.2)	36 (8.8)
	Two	21 (5.1)	57 (13.9)
	Three	27 (6.6)	84 (20.5)
	Four	30 (7.3)	114 (27.8)
	Five	57 (13.9)	171 (41.7)
	Six	64 (15.6)	235 (57.3)
	Seven	175 (42.7)	410 (100)
Number of identified consequences of stroke	Zero	20 (4.9)	20 (4.9)
	One	5 (1.2)	25 (6.1)
	Two	21 (5.1)	46 (11.2)
	Three	56 (13.7)	102 (24.9)
	Four	87 (21.2)	189 (46.1)
	Five	221 (53.9)	410 (100)

Most participants (86.3%) stated that they would transport patients with suspected stroke (based on symptoms) to hospital, while 7.3% did not know what to do in case of stroke. In general, when people were asked about rating their knowledge about stroke, more than three-quarters of the participants (78.5%) reported they had general knowledge about the stroke, while the rest (21.5%) either reported knowing the name of the disease or having no knowledge of the disease. Finally, most of the participants (94.6%) would like more information about stroke.

**Figure 1:**
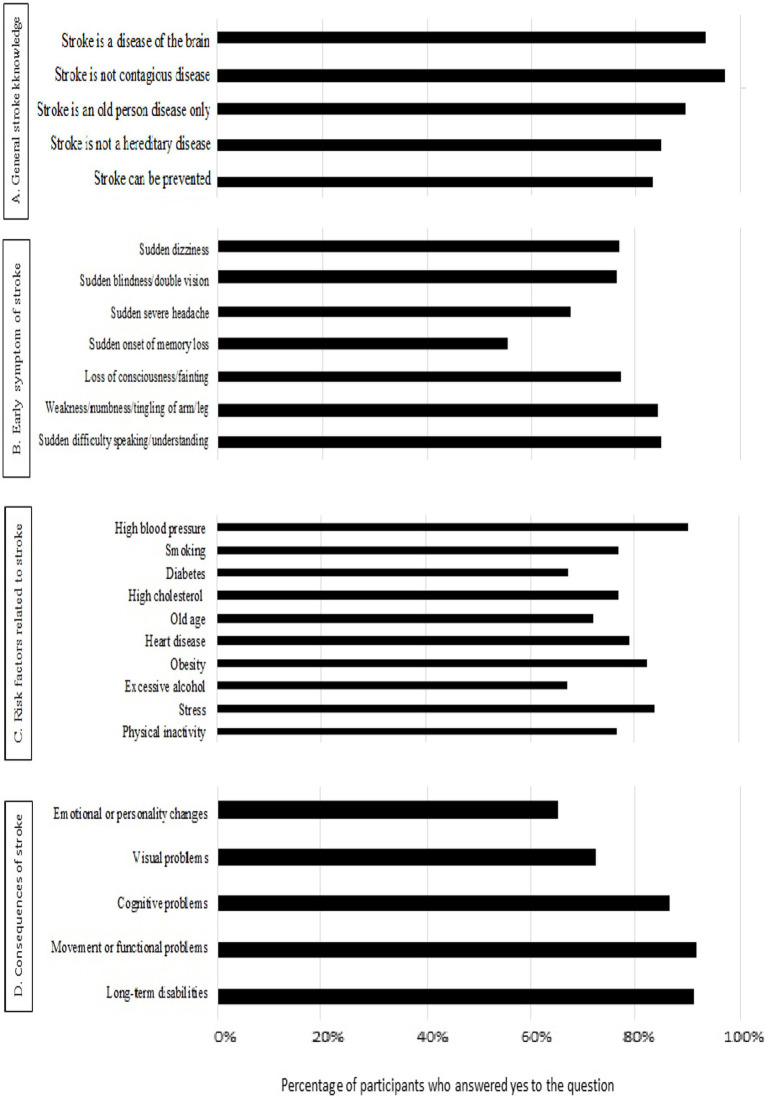
Knowledge of Sudanese participants (*n* = 410) related to **(A)** Stroke, **(B)** Early symptoms of stroke, **(C)** Risk factors related to stroke, and **(D)** Consequences of stroke.

Figure 1 presents the knowledge of Sudanese participants related to different issues about the stroke. For general stroke knowledge (Figure 1A), the majority of the participants were aware that stroke is not a contagious disease (96.8%), it is affecting the brain (93.2%), and it can be preventable (83.4%). For early symptoms of stroke (Figure 1B), most of the respondents answered that the two main early symptoms of stroke were sudden difficulty speaking, and weakness, numbness, and/or tingling of arm and leg, with 84.9, and 84.4%, respectively. For the stroke related risk factors (Figures1C), most of the participants (90.2%) believed that hypertension was the most common risk factor of stroke, followed by psychological stress (83.9%) and obesity (82.4%). Finally, knowledge related to consequences of stroke (Figure 1D), most of the participants reported that stroke can cause movement/functional problems and long-term disabilities with 91.7, and 91.2%, respectively.

Although the sample showed a variable level of knowledge about stroke, only females were significantly more able to list at least one correct risk factors related to stroke compared to males (99.5% versus 93.5%, *p* = 0.001). There was no other significant relationships between the sociodemographic characteristics or past medical history with risk factors, early symptoms and consequences of stroke (Table 3).

**Table 3:** Association of risk factors, early symptoms and consequences of stroke with the sociodemographic characteristics and past medical history (*n* = 410).

Variables		Risk factor(s) identified (≥1)			Early symptom(s) identified (≥1)			Consequence(s) identified (≥1)		
		Yes (*n* = 395) *n* (%)	No (*n* = 15) *n* (%)	*p*-value	Yes (*n* = 379) *n* (%)	No (*n* = 31) *n* (%)	*p*-value	Yes (*n* = 390) *n* (%)	No (*n* = 20) *n* (%)	*p*-value
Socio-demographic characteristics										
Gender	Male	201 (93.5)	14 (6.5)	**0.001**	196 (91.2)	19 (8.8)	0.352	201 (93.5)	14 (6.5)	0.115
	Female	194 (99.5)	1 (0.5)		183 (93.8)	12 (6.2)		189 (96.9)	6 (3.1)	
Age groups (years)	Less than 30 years	146 (98.0)	3 (2.0)	0.276	142 (95.3)	7 (4.7)	0.095	143 (96.0)	6 (4.0)	0.604
	Between 30 and 49 years	191 (96.0)	8 (4.0)		178 (89.4)	21 (10.6)		187 (94.0)	12 (6.0)	
	More than 50 years	58 (93.5)	4 (6.5)		59 (95.2)	3 (4.8)		60 (96.8)	2 (3.2)	
Residence area	Urban	370 (96.6)	13 (3.4)	0.259	354 (92.4)	29 (7.6)	1	305 (95.3)	18 (4.7)	0.633
	Rural	25 (92.6)	2 (7.4)		25 (92.6)	2 (7.4)		25 (92.6)	2 (7.4)	
Marital status	Single	174 (96.1)	7 (3.9)	1	166 (91.7)	15 (8.3)	0.968	171 (94.5)	10 (5.5)	0.932
	Married	202 (96.2)	8 (3.8)		194 (92.4)	16 (7.6)		200 (95.2)	10 (4.8)	
	Divorced/Widowed	19 (100)	0 (0)		19 (100)	0 (0)		19 (100)	0 (0)	
Educational level	School	29 (93.5)	2 (6.5)	0.316	30 (96.8)	1 (3.2)	0.495	30 (96.8)	1 (3.2)	1
	University	336 (96.6)	13 (3.4)		349 (92.1)	30 (7.9)		360 (95.0)	19 (5.0)	
Employment status	Unemployed	115 (98.3)	2 (1.7)	0.250	109 (93.2)	8 (6.8)	0.838	112 (95.7)	5 (4.3)	0.805
	Employed	280 (95.6)	13 (4.4)		270 (92.2)	23 (7.8)		278 (94.9)	15 (5.1)	
Income level	Low	72 (97.3)	2 (2.7)	0.889	71 (95.9)	3 (4.1)	0.400	71 (95.9)	3 (4.1)	0.515
	Medium	150 (96.2)	6 (3.8)		144 (92.3)	12 (7.7)		146 (93.6)	10 (6.4)	
	High	173 (96.1)	7 (3.9)		164 (91.1)	16 (8.9)		173 (96.1)	7 (3.9)	
Smoking status	No	244 (96.8)	8 (3.2)	0.592	230 (91.3)	22 (8.7)	0.338	240 (95.2)	12 (4.8)	1
	Yes	151 (95.6)	7 (4.4)		149 (94.3)	9 (5.7)		150 (94.9)	8 (5.1)	
Past medical history										
Hypertension	No	287 (95.7)	13 (4.3)	0.373	275 (91.7)	25 (8.3)	0.403	284 (94.7)	16 (5.3)	1
	Yes	108 (98.2)	2 (1.8)		104 (94.5)	6 (5.5)		106 (96.4)	4 (3.6)	
Diabetes Mellitus	No	325 (95.9)	14 (4.1)	0.532	312 (92.0)	27 (8.0)	0.610	323 (95.3)	16 (4.7)	1
	Yes	70 (98.6)	1 (1.4)		67 (94.4)	4 (5.6)		67 (94.4)	4 (5.6)	
Dyslipidemia	No	315 (95.7)	14 (4.3)	0.322	301 (91.5)	28 (8.5)	0.166	312 (94.8)	17 (5.2)	0.776
	Yes	80 (98.8)	1 (1.2)		78 (96.3)	3 (3.7)		78 (96.3)	3 (3.7)	
Heart diseases	No	339 (96.0)	14 (4.0)	0.705	324 (91.8)	29 (8.2)	0.285	334 (94.6)	19 (5.4)	0.334
	Yes	56 (98.2)	1 (1.8)		55 (96.5)	2 (3.5)		56 (98.2)	1 (1.8)	
Kidney disease	No	361 (96.0)	15 (4.0)	0.625	345 (91.8)	31 (8.2)	0.095	356 (94.7)	20 (5.3)	0.394
	Yes	34 (100)	0 (0)		34 (100)	0 (0)		34 (100)	0 (0)	
Gastro problems	No	335 (96.0)	14 (4.0)	0.709	322 (92.3)	27 (7.7)	1	331 (94.8)	18 (5.2)	0.751
	Yes	60 (98.4)	1 (1.6)		57 (93.4)	4 (6.6)		59 (96.7)	2 (3.3)	
Depression	No	358 (96.8)	12 (3.2)	0.172	344 (93.0)	2.6 (7.0)	0.209	353 (95.4)	17 (4.6)	0.430
	Yes	37 (92.5)	3 (7.5)		35 (87.5)	5 (12.5)		37 (92.5)	3 (7.5)	
Obesity	No	332 (95.7)	15 (4.3)	0.142	319 (91.9)	28 (8.1)	0.448	327 (94.2)	20 (5.8)	0.054
	Yes	63 (100)	0 (0)		60 (95.2)	3 (4.8)		63 (100)	0 (0)	

Fisher’s exact test was used when the cell counts was less than 5.Numbers in bold indicate significant *p*-values.

### Sources of information

**Figure 2:**
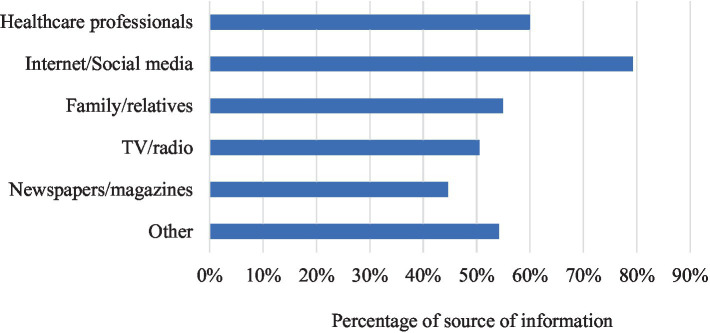
Sources of information about stroke as reported by Sudanese respondents (*n* = 410).

Figure 2 lists the sources of stroke information mentioned by respondents. The main source of information was internet/social media (79.3%), followed by healthcare professionals (60.0%), family and relatives (54.9%).

### Respondents’ attitudes about stroke

The majority of participants believed that stroke was a serious disease, and that patient’s life would be affected after stroke of 99.5 and 98.8%, respectively. Furthermore, the majority (96.6%) believed that family care was helpful for early recovery of patients with stroke after leaving the hospitals. However, more than a third of participants (34.4%) stated that stroke disease cannot lead to happy life of patients.

### Bivariate analysis associated with response to somebody with symptoms of stroke

Most of the participants (86.3%) reported that the first action in response to patients with stroke symptoms would be to take the patient directly to a hospital. There were no significant relationships between all sociodemographic characteristics, or past medical history with respondents reactions to a patient with stroke (Table 4).

**Table 4:** Association of taking a patient who is experiencing stroke to the hospital with sociodemographic characteristics, and past medical history (*n* = 410).

Variables		Taking a patient who is experiencing stroke to the hospital		
		Yes (*n* = 354) *n* (%)	No (*n* = 56) *n* (%)	*p*-value
Sociodemographic characteristics				
Gender	Male	181 (84.2)	34 (15.8)	0.197
	Female	173 (88.7)	22 (11.3)	
Age group (years)	Less than 30 years	133 (89.3)	16 (10.7)	0.255
	Between 30 and 49 years	166 (83.4)	33 (16.6)	
	More than 50 years	55 (88.7)	7 (11.3)	
Residence area	Urban	332 (86.7)	51 (13.3)	0.349
	Rural	22 (81.5)	5 (18.5)	
Marital status	Single	157 (86.7)	24 (13.3)	0.853
	Married	179 (85.2)	31 (14.8)	
	Divorced/Widowed	18 (94.7)	1 (5.3)	
Educational level	School	26 (83.9)	5 (16.1)	0.595
	University	328 (86.5)	51 (3.5)	
Employment status	Unemployed	101 (86.3)	16 (13.7)	1
	Employed	253 (86.3)	40 (13.7)	
Income level	Low	64 (86.5)	10 (13.5)	0.688
	Medium	132 (84.6)	24 (15.4)	
	High	158 (87.8)	22 (12.2)	
Smoking status	No	215 (85.3)	37 (14.7)	0.465
	Yes	139 (88.0)	19 (12.0)	
Past medical history				
Hypertension	No	258 (86.0)	42 (14.0)	0.751
	Yes	96 (87.3)	14 (12.7)	
Diabetes mellitus	No	292 (86.1)	47 (13.9)	0.852
	Yes	62 (87.3)	9 (12.7)	
Dyslipidemia	No	258 (86.6)	44 (13.4)	0.857
	Yes	69 (85.2)	12 (14.8)	
Heart diseases	No	303 (85.8)	50 (14.2)	0.539
	Yes	51 (89.5)	6 (10.5)	
Kidney disease	No	322 (85.6)	54 (14.4)	0.202
	Yes	32 (94.1)	2 (5.9)	
Gastro problems	No	52 (85.2)	47 (13.5)	0.840
	Yes	52 (85.2)	9 (14.8)	
Depression	No	318 (85.9)	52 (14.1)	0.630
	Yes	36 (90.0)	4 (10.0)	
Obesity	No	297 (85.6)	50 (14.4)	0.329
	Yes	57 (90.5)	6 (9.5)	

Fisher’s exact test was used when the cell counts was less than 5.

### Multivariable analysis associated with stroke knowledge

In the logistic regression analysis, when considering identification of at least a risk factor as the dependent variable, the multivariable analysis showed that females compared to males and patients with depression versus without depression had significantly higher odds to identify at least a risk factor (OR of 14.716 [95% CI 1.901;113.908] and 0.241 [95% CI 0.059; 0.984], respectively). Table 5 shows the results of multivariate analysis.

**Table 5:** Multivariate analysis associated with stroke knowledge among the participants (*n* = 410).

Variables	*β* (SE)	OR (95% CI)	*p*-value
Risk factor(s) identified (≥1)			
Gender (female versus male*)	2.689 (1.044)	14.716 (1.901;113.908)	**0.01**
Depression (yes versus no*)	−1.421 (0.717)	0.241 (0.059; 0.984)	**0.047**
Consequence(s) identified (≥1)			
Gender (female versus male*)	0.829 (0.5)	2.3 (0.6; 8.4)	0.214

*β*, Beta; SE, standard error; OR, adjusted ratio; CI, confidence interval.

Logistic regression taking identification of stroke risk factors, stroke early symptoms, stroke consequences as the dependent variables and sociodemographic factors (gender, age, depression, dyslipidemia, kidney disease, and obesity) as independent variables. Numbers in bold indicate significant *p*-values. *Stands for the reference category.

## Discussion

The present study aimed at examining factors associated with stroke knowledge and awareness among the general population of Sudan. It is extremely important to prevent stroke and initiate early and timely management through assessing people’s knowledge and awareness of risk factors and their modifications, as well as their warning symptoms and management. Therefore, creating effective educational and preventative measures are crucial for assessing the factors associated with stroke knowledge among different sociodemographic groups within communities.

The results indicated that around half of the sample were females, and aged between 30 and 49 years old. Moreover, more than half of the participants have low- or medium-monthly incomes. In addition, the majority of the participants have university education, with more than two-thirds of them were employed. Most of the participants had heard of the condition previously and reported that stroke is a disease of brain, and it is a serious disease. Overall, participants’ general knowledge about stroke was insufficient, with only about a third of participants correctly identifying all risk factors, all consequences, and early symptoms of stroke.

Previous studies reported suboptimal in different communities regarding strokes, even among those who had experienced it before (17–19). A recent study in Sudan reported a relatively low mean awareness score among Sudanese people (20). In addition, the mean awareness score was found to be statistically associated with the level of education (20). According to our study findings, a variable level of knowledge about stroke was found among participants. The participants in our study expressed better general knowledge about stroke compared to previous study in Sudan (20). Our findings can be interpreted by the fact that the majority of our participants had university educational level which could have raised the level of knowledge. Additionally, most of our participants identified at least one risk factor, one symptom related to stroke and one consequence. In addition, the percentages were higher than those reported in the literature (21–23), revealing that at least one stroke risk factor may be identified by the majority of the study sample, probably as most of our study participants had higher educational levels and were employed.

As for the risk factors, there are several modifiable and non-modifiable risk factors associated with stroke, including age, race, hypertension, diabetes, and many more (24), which are becoming more prevalent among the Sudanese community according to the stepwise approach to surveillance survey of 2005 (STEPS) (25). In our study, 96.3% of 410 participants identified at least one associated risk factor for stroke compared to previous published studies in the last 3 years, which reported in the UAE (99.8%, *n* = 545), Saudi Arabia (99.5%, *n* = 398), Jordan (98.1%, *n* = 573), Lebanon (97.8%, *n* = 551), and Iraq (85.6%, *n* = 609) (8, 26–29).

Considering recent studies in the Middle East region to our study results, the aware that stroke is a type of brain disease were various among the participants with the highest in Jordan and Lebanon with more than 95.0% of the participants (28), then Iraq (92.8%) (27), Saudi Arabia (89.7%) (29), and the UAE (70.8%) (26), while the results of our studies indicated that 93.2% of the participants. In related to the participants’ awareness of stroke is preventable, the results reported by the participants were also various among the countries, with the highest in Iraq (85.6%) (27), then our Sudan sample with 83.4%, then Jordan and Saudi Arabia with 81.0% (8, 29), Lebanon (80.0%) (28), and the UAE (42.9%) (26).

A significant improvement in the rates of identified risk factors of stroke among the Sudanese community was noticed, as a previous study reported that 27.6% of their study participants did not have sufficient knowledge about stroke risk factors, contrary to our findings, where it was only 3.7% (20). According to this study, hypertension was the most reported risk factor, followed by psychological stress and obesity. Similarly, a comparative study of risk factors in young adults and older adult stroke patients in Sudan reported that arterial hypertension was the dominant risk factor among young adults and the older adult, followed by smoking (30). As well as previous studies conducted in Lebanon revealed that hypertension and psychological stress were among the most reported risk factors as well (28), in Jordan (8), Saudi (29), UAE (26), and Iraq (27).

Regarding warning signs and consequences of stroke, almost 85% reported sudden difficulty in speaking or understanding speech as the most common warning sign, followed by sudden weakness/numbness/tingling, which is similar to the findings of Lebanese and New Zealand studies (28, 31). However, a previous Sudanese survey reported paralysis of one side of the body as the most commonly identified warning symptom by 30.7%, followed by sudden difficulty in speaking or understanding by 27.1%. In comparison to the findings of the previous study, the rates of the identified consequences of stroke were significantly improved among Sudanese people. Almost 33% of the previous study participants did not know any stroke warning symptoms (20), while in our study, the percentage was only 4.9%. The improvement in the knowledge of the Sudanese community regarding risk factors, symptoms, and consequences related to stroke in our study could be attributed to the higher educational level and employment of the majority of our study participants.

Our results revealed a significant correlation between gender and knowledge about stroke risk factors, as females had better knowledge compared to males. In agreement with our study findings, other studies reported similar results (32–35). On the other hand, better knowledge of stroke warning signs among males was reported as well (8, 27). Based on the literature, the lack of consistency in the results of different studies makes it ambiguous to call for a clear association between gender differences and knowledge of stroke warning signs and risk factors (8, 27). As well as the multivariable analysis showed that patients with no depression had a significant correlation in identifying stroke risk factors, early stroke symptoms, and stroke consequences. A previous study revealed a significant inverse correlation between depression and knowledge. It is reported that depressed patients had poorer knowledge and a decline in knowledge as depression increases, suggesting that cognitive function may be impaired by depression (36, 37).

### Strengths and limitations

There are some limitations to this study that can be identified. First, the study utilized an online survey that mandates technical requirements and access to internet and mobile/computer and reading ability, hence the population in this study does not represent the whole population in Sudan. Second, information bias connected to on-demand resource accessibility can jeopardize answer credibility. Third, selection bias associated with the snowball collection technique could be an issue, as there is no guarantee for random selection. Furthermore, there is a high percentage of missing data that leads to big odds ratio. On the other hand, our study has several strengths as it added data to the literature about stroke disease awareness which highlighted the lack of knowledge regarding stroke among the Sudanese population.

## Conclusion

In general, the Sudanese community demonstrated suboptimal knowledge regarding stroke risk factors, early symptoms, and consequences. Females and individuals without a medical history of depression exhibited higher knowledge scores. Recognition of specific aspects varied, with social media serving as a prominent information source. Tailored interventions focusing on individuals with inadequate stroke literacy are needed to improve stroke awareness. Further studies, more representative of the general Sudanese population and with a larger sample size, are necessary to confirm our findings.

## Data availability statement

The original contributions presented in the study are included in the article/supplementary material, further inquiries can be directed to the corresponding author.

## Ethics statement

The studies involving humans were approved by the research ethics committee of the Faculty of Pharmacy, Al Neelain University, Khartoum, Sudan (NPH1021). The studies were conducted in accordance with the local legislation and institutional requirements. The participants provided their written informed consent to participate in this study.

## Author contributions

EE: Writing – review & editing, Writing – original draft. FJ: Writing – original draft, Writing – review & editing, Conceptualization, Methodology. DS: Methodology, Writing – original draft. HaA: Writing – original draft. MA: Writing – original draft, Writing – review & editing. ZKha: Methodology, Writing – original draft. YA: Writing – original draft, Writing – review & editing. MB: Writing – original draft, Writing – review & editing. ZKhi: Writing – original draft. HuA: Writing – original draft. NM: Investigation, Writing – original draft. SC: Formal analysis, Writing – original draft. SK: Writing – review & editing. SH: Formal analysis, Writing – review & editing. DM: Writing – review & editing, Conceptualization, Data curation, Methodology, Supervision. HH: Writing – review & editing.
